# Applicability and Risk Stratification of QRISK®, Framingham Risk Score, Systematic Coronary Risk Evaluation (SCORE), and American College of Cardiology/American Heart Association (ACC/AHA) Risk Assessment Tools Among Patients in Taif, Saudi Arabia

**DOI:** 10.7759/cureus.86910

**Published:** 2025-06-28

**Authors:** Suha A Alnefaie, Ameerah S Bajaber, Ahmed S Alzahrani, Rakan S Alotaibi, Amjad J Alotaibi

**Affiliations:** 1 Preventive Medicine, Alhada Armed Forces Hospital, Al Hada, SAU; 2 Internal Medicine, Alhada Armed Forces Hospital, Al Hada, SAU; 3 Preventive Medicine, Armed Forces Hospital, Madina, SAU

**Keywords:** ascvd risk estimator, cad, cardiovascular disease, coronary artery disease, cvd, framingham risk score, qrisk®, risk assessment tools, saudi arabia, score

## Abstract

Introduction

Cardiovascular disease (CVD) is the leading cause of death globally and continues to pose significant challenges to public health in Saudi Arabia. Risk prediction tools are essential in guiding preventive strategies, yet their accuracy may vary across populations. This study aimed to assess and compare the applicability, which was based on the availability of all required variables, and the risk stratification performance of four cardiovascular risk assessment tools, such as the Framingham Risk Score (FRS), American College of Cardiology/American Heart Association (ACC/AHA) Atherosclerotic Cardiovascular Disease (ASCVD) Risk Estimator, Systematic Coronary Risk Evaluation (SCORE), and QRISK®, among Saudi patients diagnosed with acute coronary syndrome (ACS).

Methods

A retrospective cross-sectional study was conducted at Alhada Armed Forces Hospital in Taif, Saudi Arabia, including all Saudi patients aged 18 years and older who were diagnosed with either a first or second myocardial infarction (MI) between January 2020 and January 2025. Data were collected through patient interviews and electronic medical records to retrospectively reconstruct the cardiovascular risk profile prior to the first MI event. Risk scores were calculated using the four tools, and each tool's applicability was based on the availability of all required variables. Statistical analyses were performed to assess tool applicability, risk stratification, and correlations among the tools.

Results

A total of 65 patients were included, with a mean age of 61 ± 13 years and a mean BMI of 29.9 ± 4.5 kg/m². The majority were male (76.9%), and common comorbidities included hypertension (49.2%), chronic kidney disease (10.8%), and atrial fibrillation (12.3%). QRISK® had the highest applicability (98.5%), followed by FRS (86.2%), ACC/AHA ASCVD Risk Estimator (73.8%), and SCORE (66.2%). SCORE identified the highest proportion of high-risk patients (24.3%), followed by QRISK® (21.6%) and FRS (13.5%). Strong-to-very strong correlations were observed between the tools, especially between QRISK® and ACC/AHA ASCVD Risk Estimator (r = 0.937) and QRISK® and FRS (r = 0.905).

Conclusion

QRISK® showed the highest applicability in this cohort; however, applicability does not reflect predictive accuracy. As these tools were designed for primary prevention, their use in post-MI patients may overestimate risk. Variability in high-risk classifications by the tools could affect preventive decisions. These findings underscore the need for further validation in larger, prospective, primary prevention studies within the Saudi population.

## Introduction

Cardiovascular disease (CVD) ranks as the leading cause of death globally [1]. In Saudi Arabia, CVD accounts for approximately 42% of all deaths [2], and over 30% of adults aged 18 and above are estimated to be at risk of experiencing a cardiovascular event [3,4]. This high burden highlights the importance of accurate risk assessment tools to guide prevention strategies and reduce the incidence of CVD in the Saudi population.

Risk assessment tools enable healthcare providers to estimate an individual’s probability of developing cardiovascular events, allowing for early interventions tailored to specific risk profiles [5,6]. Among the most widely used tools are the Framingham Risk Score (FRS), the Systematic Coronary Risk Evaluation (SCORE), the Atherosclerotic Cardiovascular Disease (ASCVD) Risk Estimator by the American College of Cardiology/American Heart Association (ACC/AHA), and the QRISK3 tool developed in the UK [7-10]. The FRS estimates the 10-year risk of coronary heart disease using several risk factors [7]. The SCORE, developed by the European Society of Cardiology, estimates fatal and non-fatal cardiovascular events [8]. The ASCVD Risk Estimator, introduced in 2013 by the ACC/AHA, integrates data from major cohort studies like the Framingham and Atherosclerosis Risk in Communities (ARIC) and is used extensively in clinical guidelines for cardiovascular risk prediction [9]. Meanwhile, QRISK3, updated regularly and recommended by the UK’s NICE guidelines, includes a broader set of risk factors such as family history of CVD, mental illness, chronic kidney disease, corticosteroid use, and migraine [10].

The performance of the widely used cardiovascular risk assessment tools may vary across populations, as they were not developed or validated for the Saudi population, whose demographic and clinical characteristics differ from those of the original cohorts [11]. This highlights the necessity of local validation to assess their predictive accuracy in Saudi clinical practice.

Several international and local studies have attempted to compare the performance of these tools. A cross-sectional study in India found that the British Joint Societies risk calculator identified the highest percentage of patients at high risk, while the WHO charts significantly underestimated risk [12]. In the UK, QRISK2 outperformed the FRS equation in identifying patients at high 10-year risk for CVD [13]. A 2022 scoping review concluded that QRISK was the most accurate among various calculators across populations, while WHO scores were the least accurate [14]. In Jeddah, Saudi Arabia, a cross-sectional study found QRISK® practical and applicable, whereas the ACC/AHA score had superior predictive accuracy for identifying high-risk individuals [15]. Similarly, a retrospective study in Tabuk indicated that the ACC/AHA estimator performed well in East Mediterranean populations, but not for South Asians [16]. Although the Saudi Heart Association currently recommends using the FRS or SCORE systems for clinical assessment, this recommendation lacks direct evidence supporting their applicability to the Saudi population. In fact, one local study indicated that QRISK® had better predictive accuracy for CVD in Saudi individuals compared to other international tools [17].

Therefore, this study aims to assess and compare the applicability, which was based on the availability of all required variables, and the risk stratification performance of four widely used cardiovascular risk assessment tools, such as the FRS, ASCVD Risk Estimator, SCORE2, and QRISK3, among Saudi patients with acute coronary syndrome (ACS), including those with myocardial infarction (MI), using retrospectively reconstructed pre-event data in Alhada Armed Forces Hospital, Taif.

## Materials and methods

Study design and setting

This retrospective cross-sectional analytical study was conducted at the Cardiac Care Unit (CCU) of Alhada Armed Forces Hospital in Taif, Saudi Arabia, from January 1, 2020, to January 1, 2025.

Study population and sampling

A consecutive sampling technique was used to select eligible patients. The inclusion criteria were adult patients (aged ≥18 years) admitted with a diagnosis of ACS, including both first and second MI events. Patients admitted for non-ACS cardiac conditions (e.g., heart failure without ischemia, arrhythmias) were excluded.

Data collection

Data were collected through direct patient interviews and a review of the hospital’s electronic health records (Wipro system) to retrospectively reconstruct the cardiovascular risk profile prior to the first MI event. A structured data collection sheet was developed to gather demographic, clinical, and laboratory information required for cardiovascular risk assessment.

The collected variables included age, gender, ethnicity, height, weight, systolic blood pressure, total cholesterol, high-density lipoprotein (HDL) cholesterol, smoking status, diabetes mellitus, rheumatoid arthritis, chronic kidney disease, atrial fibrillation, family history of premature cardiovascular disease in a first-degree relative, and use of antihypertensive medication.

Risk score calculation

Cardiovascular risk scores were calculated for each patient using four validated tools: the FRS for hard coronary heart disease (FRS-hCHD), SCORE (HeartScore Europe very high risk), ASCVD Risk Estimator (ASCVD Risk Estimator Plus), and QRISK (QRISK®3-2018). Calculations were performed using the respective published algorithms and online calculators when applicable. Applicability was defined as the ability to compute the risk score based on the availability of all required input variables for a given patient.

The FRS estimates the 10-year risk of cardiovascular events using age, sex, cholesterol levels, blood pressure, smoking, and diabetes. The ACC/AHA ASCVD Risk Estimator calculates the 10-year risk of ASCVD including MI and stroke, using age, sex, race, cholesterol, blood pressure, diabetes, and smoking. SCORE2 predicts the 10-year risk of cardiovascular morbidity and mortality in European populations using age, sex, systolic blood pressure, non-HDL cholesterol, and smoking. QRISK3, a UK-based model, estimates the 10-year risk of CVD and incorporates a broader range of variables, including BMI, ethnicity, family history, atrial fibrillation, chronic kidney disease, and other comorbidities.

Risk stratification was based on each tool’s recommended thresholds for high cardiovascular risk. Patients were categorized as high-risk if their 10-year estimated risk met or exceeded the cutoff defined by each model: ≥20% for FRS, ASCVD, and QRISK3 and ≥10% for SCORE2.

Statistical analysis

The data were analyzed using R statistical software version 4.4.2 (R Foundation for Statistical Computing, Vienna, Austria, https://www.R-project.org/). Continuous variables were expressed as mean ± standard deviation, while categorical variables were expressed as frequency and percentage. Descriptive statistics were used to analyze the baseline characteristics of the study population. The risk estimates derived from the different risk scores were compared using Wilcoxon's signed-rank test for the non-dichotomized risk scores or McNemar's test when the risk scores were dichotomized as <20% or ≥20%. Pearson’s correlation coefficient (r) was estimated to assess the relationship between various risk score calculators. p < 0.05 was considered statistically significant. Missing values and "not applicable" were excluded from the analyses to maintain the consistency and accuracy of the study results.

## Results

Our study included 65 Saudi patients with a mean age of 61 ± 13 years and a mean BMI of 29.9 ± 4.5 kg/m². The majority of the study population were males (76.9%). Among the patients, 20.0% were current smokers and 26.2% were former smokers. Hypertension was present in 49.2% of patients, 10.8% had chronic kidney disease, and 12.3% had atrial fibrillation. Only seven patients reported a family history of coronary artery stenosis (CAS) in a first-degree relative under 60 years of age.

The population had a mean systolic blood pressure of 129 ± 22 mmHg and a mean diastolic blood pressure of 75 ± 12 mmHg. The mean HDL cholesterol was 1.02 ± 0.41 mmol/L, and the mean total cholesterol was 4.63 ± 1.23 mmol/L (Table 1).

**Table 1:** Baseline characteristics of the study participants ^1^Mean ± SD; n (%).

Characteristics	N = 65^1^
Age (mean)	61 ± 13
Gender (n, %)	
Female	15.0 (23.1%)
Male	50.0 (76.9%)
High cholesterol (n, %)	
No	52.0 (80.0%)
Yes	13.0 (20.0%)
Hypertension (n, %)	
No	33.0 (50.8%)
Yes	32.0 (49.2%)
DMT1 (n, %)	
No	65.0 (100.0%)
Smoker (n, %)	
No	52.0 (80.0%)
Yes	13.0 (20.0%)
Former smoker (n, %)	
No	48.0 (73.8%)
Yes	17.0 (26.2%)
Never smoker (n, %)	
No	30.0 (46.2%)
Yes	35.0 (53.8%)
Coronary artery stenosis (CAS) in a first-degree relative under the age of 60 (n, %)	
No	56.0 (86.2%)
Not known	2.0 (3.1%)
Yes	7.0 (10.8%)
Chronic kidney disease (CKD) (n, %)	
No	58.0 (89.2%)
Yes	7.0 (10.8%)
Headache, migraine (n, %)	
No	61.0 (93.8%)
Yes	4.0 (6.2%)
Insomnia (n, %)	
No	58.0 (89.2%)
Yes	7.0 (10.8%)
Atrial fibrillation (n, %)	
No	57.0 (87.7%)
Yes	8.0 (12.3%)
Multiple sclerosis (MS) (n, %)	
No	65.0 (100.0%)
Systemic lupus erythematosus (SLE) (n, %)	
No	65.0 (100.0%)
Erectile dysfunction (n, %)	
No	47.0 (72.3%)
Not applicable	15.0 (23.1%)
Yes	3.0 (4.6%)
Steroid use (n, %)	
No	64.0 (98.5%)
Yes	1.0 (1.5%)
Depression (n, %)	
No	63.0 (96.9%)
Yes	2.0 (3.1%)
Schizophrenia (n, %)	
No	65.0 (100.0%)
Bipolar disorder (n, %)	
No	65.0 (100.0%)
Atypical selective serotonin reuptake inhibitor (SSRI) (n, %)	
No	65.0 (100.0%)
Systolic blood pressure (SBP) (mean)	129 ± 22
Diastolic blood pressure (DBP) (mean)	75 ± 12
Body mass index (BMI) (mean)	29.9 ± 4.5
High-density lipoprotein (HDL) (mean)	1.02 ± 0.41
Total cholesterol (mean)	4.63 ± 1.23

The 10-year cardiovascular risk scores’ applicability, defined as the proportion of patients for whom all required variables were available, varied significantly across assessment tools: SCORE (66.2%), ACC/AHA ASCVD Risk Estimator (73.8%), FRS (86.2%), and QRISK® (98.5%) (Table 2). Some patients were not eligible for scoring due to factors such as age, blood pressure, or lipid values falling outside the tools’ validated input ranges.

**Table 2:** Cardiovascular risk level applicability SCORE: Systematic Coronary Risk Evaluation, FRS: Framingham Risk Score, ACC/AHA ASCVD: American College of Cardiology/American Heart Association Atherosclerotic Cardiovascular Disease.

Cardiovascular risk level applicability	QRISK®	SCORE	FRS	ACC/AHA ASCVD Risk Estimator
Applicable	64.0 (98.5%)	43.0 (66.2%)	56.0 (86.2%)	48.0 (73.8%)
Not applicable	1.0 (1.5%)	22.0 (33.8%)	9.0 (13.8%)	17.0 (26.2%)

Although QRISK® demonstrated the highest applicability, this reflects broader data availability rather than superior predictive accuracy and should be interpreted with caution.

**Figure 1:**
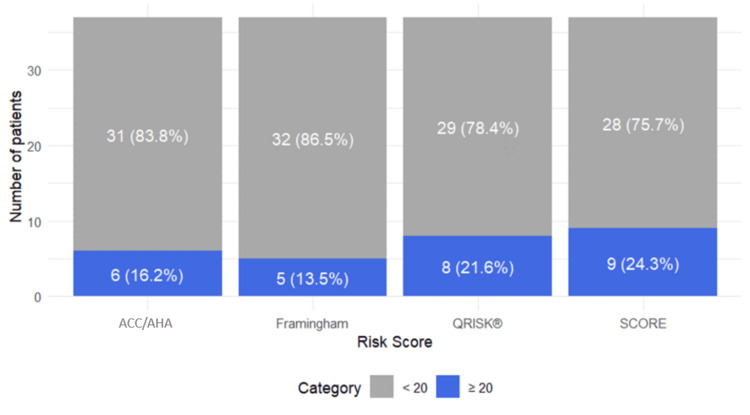
Ten-year dichotomized risk categories of various risk score calculators ACC/AHA: American College of Cardiology/American Heart Association, SCORE: Systematic Coronary Risk Evaluation.

Based on the published thresholds (≥20% for QRISK®, Framingham, and ACC/AHA; ≥10% for SCORE2), the highest proportion of patients classified as high-risk was identified by SCORE2 (24.3%), followed by QRISK® (21.6%), and Framingham (13.5%) (Figure 1, Table 3).

**Table 3:** Ten-year dichotomized risk categories SCORE: Systematic Coronary Risk Evaluation, FRS: Framingham Risk Score, ACC/AHA ASCVD: American College of Cardiology/American Heart Association Atherosclerotic Cardiovascular Disease.

10-year risk	QRISK®	SCORE	FRS	ACC/AHA ASCVD Risk Estimator
<20%	29.0 (78.4%)	28.0 (75.7%)	32.0 (86.5%)	31.0 (83.8%)
≥20%	8.0 (21.6%)	9.0 (24.3%)	5.0 (13.5%)	6.0 (16.2%)

The Wilcoxon signed-rank test revealed statistically significant differences in the mean 10-year CVD risk scores across most tools (p < 0.001), except between QRISK® and SCORE2, and between FRS and ACC/AHA ASCVD Risk Estimator (Table 4).

**Table 4:** Comparison of the 10-year cardiovascular disease (CVD) risk scores The p-value was calculated using Wilcoxon's signed-rank test. SCORE: Systematic Coronary Risk Evaluation, FRS: Framingham Risk Score, ACC/AHA ASCVD: American College of Cardiology/American Heart Association Atherosclerotic Cardiovascular Disease.

	QRISK®	SCORE	FRS
SCORE	0.2853		
FRS	0.0001	<0.0001	
ACC/AHA ASCVD Risk Estimator	<0.0001	<0.0001	0.9219

Using the McNemar's test, no significant differences were found in the proportions of patients categorized into high-risk (≥20%) versus low-risk (<20%) across the tools (Table 5).

**Table 5:** Comparison of the 10-year dichotomized risk categories The p-value was calculated using the McNemar's test. SCORE: Systematic Coronary Risk Evaluation, FRS: Framingham Risk Score, ACC/AHA ASCVD: American College of Cardiology/American Heart Association Atherosclerotic Cardiovascular Disease.

	QRISK®	SCORE	FRS
SCORE	>0.9		
FRS	0.3711	0.2888	
ACC/AHA ASCVD Risk Estimator	0.4795	0.2482	>0.9

Pearson correlation analysis was conducted to evaluate the strength of association between risk scores. Very strong correlations were observed between QRISK® and FRS (r = 0.905), QRISK® and ACC/AHA ASCVD Risk Estimator (r = 0.937), and ACC/AHA ASCVD Risk Estimator and both FRS (r = 0.914) and SCORE2 (r = 0.913). Strong correlations were also noted between QRISK® and SCORE2 (r = 0.807) and FRS and SCORE2 (r = 0.724) (Table 6). These correlations reflect the linear relationship between scores but do not indicate agreement or predictive equivalence.

**Table 6:** Correlation among risk score instruments SCORE: Systematic Coronary Risk Evaluation, FRS: Framingham Risk Score, ACC/AHA ASCVD: American College of Cardiology/American Heart Association Atherosclerotic Cardiovascular Disease.

	QRISK®	SCORE	FRS	ACC/AHA ASCVD Risk Estimator
QRISK®	1.000	0.807	0.905	0.937
SCORE	0.807	1.000	0.724	0.913
FRS	0.905	0.724	1.000	0.914
ACC/AHA ASCVD Risk Estimator	0.937	0.913	0.914	1.000

Patients for whom a risk score could not be calculated due to missing or inapplicable variables were excluded from each respective comparison and correlation analysis.

## Discussion

This study evaluated the applicability and risk stratification of four commonly used cardiovascular risk assessment tools, such as QRISK®, ACC/AHA ASCVD Risk Estimator, FRS, and SCORE, for forecasting cardiovascular risk in a sample of 65 Saudi patients experiencing their first and second MI events. The variability among both the applicability and outcomes of these tools highlights the need to adopt the most suitable tool tailored to the demographics of this population.

Among the four tools, QRISK® demonstrated the highest applicability rate (98.5%), primarily due to its broad inclusion of many variables such as ethnicity, comorbidities, and lifestyle factors (10). However, the QRISK® tool has not been validated for the Saudi population, and its estimates may not fully reflect the local epidemiological profile. Contrarily, the SCORE risk calculator had the lowest applicability (66.2%), while the FRS and ACC/AHA ASCVD Risk Estimator were applicable in 86.2% and 73.8% of patients, respectively. Despite its lower applicability, the SCORE risk calculator identified the maximum number of patients with high CVD risk (≥20%).

In terms of other scores, the lack of ethnicity considerations and age limitations to ≤79 years in the ACC/AHA ASCVD Risk Estimator and ≥40 years in the SCORE limit the prediction for very old and middle-aged patients, respectively. Furthermore, the SCORE tool's limitations in systolic blood pressure ≥ 100 and cholesterol levels ≥ 3 mmol/L restrict its applicability to a wide category of patients, which explains why SCORE was the least applicable instrument in our study [18,19].

Our findings coincide with the results from a previous study conducted on the Saudi population by Hasabullah et al., who reported comparable applicability rates with QRISK® (95.3%) and SCORE (22.5%) [15]. Meanwhile, they reported that the ACC/AHA ASCVD Risk Estimator** **was able to identify the highest proportion of high-risk populations (66.7%) compared to other tools [15]. Another Saudi study compared cardiac risk scores among the East Mediterranean and South Asian populations using four risk assessment instruments (ACC/AHA, Framingham, WHO/ISH, and SCORE) and found that the ACC/AHA tool demonstrated the highest ability to forecast high-risk (>10%) in more than half of the study population, especially the East Mediterranean population, followed by Framingham, WHO/ISH, and SCORE. However, they concluded the need for a population tool for South Asians to evaluate the accurate CVD risk [16]. This study reported different outcomes compared to our findings, probably due to enrolling demographically different study populations. However, most of these comparative studies were conducted in primary prevention settings, whereas our study applied these tools retrospectively in post-MI patients, which may partially explain the difference in high-risk classification rates.

Similar studies were conducted in different geographical regions. A study enrolling Indian population comparing the accuracy of four risk scores in evaluating cardiovascular risk (FRS, WHO risk prediction charts, ACC/AHA pooled cohort equations, and the 3rd Joint British Societies (JBS) risk calculator) demonstrated that the JBS risk calculator identified the highest percentage of patients at high risk (≥20%) (55.9%) compared to FRS (38.3%), ACC/AHA (30.2%), and WHO (13.4%) [12]. This finding suggests that the JBS risk calculator can be considered the most appropriate assessment tool for Indians. Garg et al. in another study enrolling Indian patients compared six risk scores and found that the FRS identified the maximum proportion of patients with high-risk CVD (>20%), followed by QRISK2, while the WHO risk calculator and ASCVD risk score calculator failed to accurately predict convenient high-risk percentages [20]. They explained the outstanding outcomes of the FRS by its highest includability of population-related variables in the score. In the same context, a prospective, cross-sectional study in the same population compared ACC/AHA, Framingham, SCORE, and QRISK3 risk calculators in patients presenting with first-time MI and concluded that QRISK3 had the maximum detection rate, whereas FRS yielded the least accurate score [21]. They consider their finding as a cornerstone of developing an Indian-specific CVD assessment risk tool. In a population-based study in Northern Iran, the ACC/AHA tool provided a relatively higher estimation of the 10-year CVD risk, compared to lower rates for the SCORE and FRS [22]. Another cross-sectional study conducted in Nigeria showed that the ACC/AHA ASCVD Risk Estimator identified half of the participants as high-risk, whereas the other two instruments classified less than 20% as high-risk [23].

The diversity of risk assessment score applicability among studies conducted in different geographical regions sheds light on the necessity of developing an ethnicity-specific assessment tool to grant maximum applicability and prediction accuracy among each specific population. Our findings may offer preliminary insights that could inform future efforts to develop a Saudi-specific cardiovascular risk assessment tool.

The strong correlations observed between tools, particularly between QRISK®, FRS, and ACC/AHA ASCVD Risk Estimator, suggest a consistent ranking of patients’ relative risk. However, correlation does not imply interchangeability or agreement in absolute risk estimates, which warrants further exploration using agreement.

This study has several limitations, including a small sample size (N = 65) and male predominance (76.9%), which may limit the generalizability and introduce sex-related bias. Moreover, the inclusion of other CVD presentations, such as stroke, would have provided a more comprehensive evaluation of risk tools. Additionally, all patients had prior MI, and applying primary prevention risk tools retrospectively in a post-MI cohort is a methodological limitation and may overestimate the risk when used in a similar cohort.

Future prospective studies with larger cohorts and equal gender enrollment in primary prevention settings are warranted to develop and validate cardiovascular risk models for the Saudi population.

## Conclusions

In this cohort, QRISK® demonstrated the highest applicability among the four cardiovascular risk assessment tools. However, applicability reflects the proportion of patients for whom a tool can be calculated and does not imply prediction accuracy. As these tools were intended for primary prevention rather than retrospective use in post-MI patients, they may overestimate risk in this group. While some tools showed strong correlations, they differed in high-risk classifications, which may impact clinical decision-making regarding preventive interventions. These findings highlight the importance of further validation through larger, prospective studies in primary prevention settings before using these tools to guide clinical decisions in the Saudi population.

## Appendices

**Table 7:** Cardiovascular risk assessment data collection sheet

Patient information	Details	
Patient name		
Medical record number		
Date of birth/age		
Gender	☐ Male ☐ Female	
Condition	Yes	No
High cholesterol	☐	☐
Hypertension	☐	☐
Type 1 diabetes	☐	☐
Current smoker	☐	☐
Former smoker	☐	☐
Never smoker	☐	☐
Number of cigarettes/day		
Years since quitting		
Coronary artery disease in a relative < age 60	☐	☐
Chronic kidney disease	☐	☐
Migraine/headache	☐	☐
Insomnia	☐	☐
Arrhythmia	☐	☐
Multiple sclerosis	☐	☐
Lupus	☐	☐
Erectile dysfunction (men only)	☐	☐
Steroid use	☐	☐
Depression (mild/moderate/severe)	☐	☐
Schizophrenia	☐	☐
Bipolar disorder	☐	☐
Use of atypical antipsychotics	☐	☐
Measurement	Value	
Systolic blood pressure (current)		
Most recent two SBP readings	______ mmHg, ______ mmHg	
Height (cm)		
Weight (kg)		
BMI		
Total cholesterol (mg/dL)		
High-density lipoprotein (HDL) (mg/dL)		
